# Pooled shRNA screen for sensitizers to inhibition of the mitotic regulator polo-like kinase (PLK1)

**DOI:** 10.18632/oncotarget.406

**Published:** 2011-12-30

**Authors:** Nancy Liu-Sullivan, Jianping Zhang, Amy Bakleh, John Marchica, Jinyu Li, Despina Siolas, Sylvie Laquerre, Yan Y. Degenhardt, Richard Wooster, Kenneth Chang, Gregory F. Hannon, Scott Powers

**Affiliations:** ^1^ Cancer Genome Center, Cold Spring Harbor Laboratory, Woodbury, NY 11797; ^2^ Cold Spring Harbor Laboratory, Cold Spring Harbor, NY 11724; ^3^ GlaxoSmithKline, Oncology Research and Development, King of Prussia, PA 19406

**Keywords:** Polo-like kinase 1, shRNA library screening, retinoids, combination therapy strategies

## Abstract

RNAi screening holds the promise of systemizing the search for combination therapeutic strategies. Here we performed a pooled shRNA library screen to look for promising targets to inhibit in combination with inhibition of the mitotic regulator polo-like kinase (PLK1). The library contained ~4,500 shRNAs targeting various signaling and cancer-related genes and was screened in four lung cancer cell lines using both high (IC_80_) and low (IC_20_) amounts of the PLK1 inhibitor GSK461364. The relative abundance of cells containing individual shRNAs following drug treatment was determined by microarray analysis, using the mock treatment replicates as the normalizing reference. Overall, the inferred influences of individual shRNAs in both high and low drug treatment were remarkably similar in all four cell lines and involved a large percentage of the library. To investigate which functional categories of shRNAs were most prominent in influencing drug response, we used statistical analysis of microarrays (SAM) in combination with a filter for genes that had two or more concordant shRNAs. The most significant functional categories that came out of this analysis included receptor tyrosine kinases and nuclear hormone receptors. Through individual validation experiments, we determined that the two shRNAs from the library targeting the nuclear retinoic acid receptor gene *RARA* did indeed silence *RARA* expression and as predicted conferred resistance to GSK461364. This led us to test whether activation of RARA receptor with retinoids could sensitize cells to GSK461364. We found that retinoids did increase the drug sensitivity and enhanced the ability of PLK1 inhibition to induce mitotic arrest and apoptosis. These results suggest that retinoids could be used to enhance the effectiveness of GSK461364 and provide further evidence that RNAi screens can be effective tools to identify combination target strategies.

## INTRODUCTION

Developing combinations of chemotherapeutic agents that increase tumor cell toxicity was a major milestone in cancer treatment [1]. Testing for advantageous combinations continues to be a driver in improving cancer care, but is limited by a lack of comprehensive methodology. RNAi screening has the potential to systematize the search for genes to target in combination with specific anti-cancer agents. Initial screens have pinpointed checkpoint kinase inhibition as an effective combination with gemcitabine treatment in pancreatic cancer cells [2], MEK inhibition as an effective combination with EGFR inhibition in *KRAS* wild-type cells pancreatic cancer cells [3], and inhibition of Wnt/Ca2+/NFAT signaling as an enhancer of BCR-ABL inhibition in CML cells [4]. Here we used RNAi screening to look for sensitizers to the candidate cancer drug GSK461364A, a potent inhibitor of polo-like kinase 1 (PLK1) [5]. PLK1 is expressed during the G2/M phase of the cell cycle and together with the Cdk1/Cdc2 kinase regulates key events in mitosis [6]. Mitotic arrest and apoptosis have been observed in preclinical studies using either RNAi, GSK461364A, or other small molecules that inhibit PLK1 [6]. Initial motivation for developing inhibitors of PLK1 as candidate cancer drugs was the potential to avoid the toxicities of traditional antimitotics that target tubulin structures equally in both cancer and nondividing cells [6, 7]. Perhaps a more compelling rationale is based on findings that PLK1 inhibition is selectively potent for cells harboring mutant *TP53* or mutant *KRAS* [8-10], which is the reverse of the usual situation where altered *TP53* and mutant *KRAS* confer drug resistance.

Several PLK1 inhibitors are in phase I or II clinical studies and some patients have achieved clinical response, although sometimes only when dosed above the maximum tolerated dose defined in the study [6]. Based on this, PLK1 inhibitors may need to be used in combination with an approved cancer drug in order to be clinically useful. In this study looked for PLK1-combination targets in non-small cell lung cancer cells (NSCLC), a clinically important tumor type that is driven to a significant degree by mutations in *TP53* and *KRAS* and that as a whole are particularly sensitive to PLK1 inhibition [7].

## RESULTS

We focused on four NSCLC cell lines, two that harbor mutant *KRAS* but are wild-type for *TP53* (A549 and NCI-H460) and two that harbor mutant *TP53* but are wild-type for *KRAS* (NCI-H522 and NCI-H322). Based on the belief that high or low concentrations of a drug could make a significant impact on the RNAi screening results, we want to screen each of the four cell lines for shRNAs that could influence the response to GSK461364A at both low and high doses (IC_20_/IC_80_). Therefore we determined the concentrations of GSK461364A that could cause 20% and 80% of maximal growth inhibition. All four cell lines were sensitive to GSK461364A, but one *TP53* mutant and one *KRAS* mutant cell line (NCI-H322 and NCI-H460) were more sensitive with IC_20_/IC_80_ values of 1 nm / 10 nM, compared to the other pair (NCI-H522 and A549), which both required higher doses to reach 20% and 80% maximal inhibition (30 nM / 100 nM).

**Figure 1:**
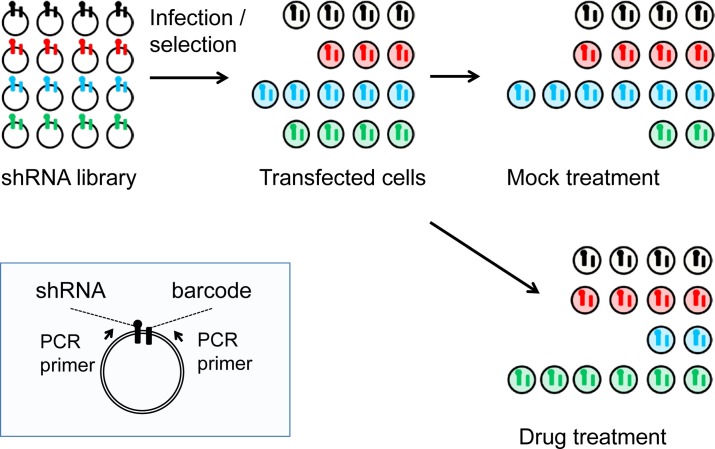
Schematic of the pooled shRNA screening methodology Shown in the blue box insert are features of the MLP retroviral vector including the two PCR primers that are used to amplify the shRNA and its linked barcode. Shown in the upper left is a representation of the relative abundance of individual shRNAs in the library as it is prepared in E. coli. Following transfection into mammalian cells and selection for stable integration, the relative abundance of individual shRNA changes, as it does following mock treatment or treatment with an inhibitory drug.

The RNAi screening methodology we employed was the pooled multiplex approach where each shRNA is tagged with a molecular barcode that together with the shRNA insert itself serve as microarray hybridization probes to deconvolute the relative abundance of the individual shRNAs (Figure 1) [11]. The 4,603-shRNA library was constructed in the retroviral vector MLP that expresses shRNAs with endogenous miR-30 flanking sequences [12]. This library targets 1,657 genes from three functional classes (kinases, cell cycle genes, functional cancer genes) with an average of 2 to 3 distinct shRNAs per gene [11]. We transfected the shRNA library into human cancer cells at a low multiplicity of infection (0.25) to ensure that each cell on average was transfected with only one distinct shRNA, and for each cell line in total we transfected twelve 150 mm. plates each containing approximately 10^7^ subconfluent cells [11]. Based on averages, each individual shRNA in the library was present in approximately 1000 cells. We selected for stable transfectants using puromycin (3- 5 days) and then used three plates to prepare genomic DNA for three biological replicates for the initial time point (t = 0). We divided the remaining nine plates into three groups; the high and low drug groups which received GSK461364A and the mock treatment group (see Methods).

**Figure 2:**
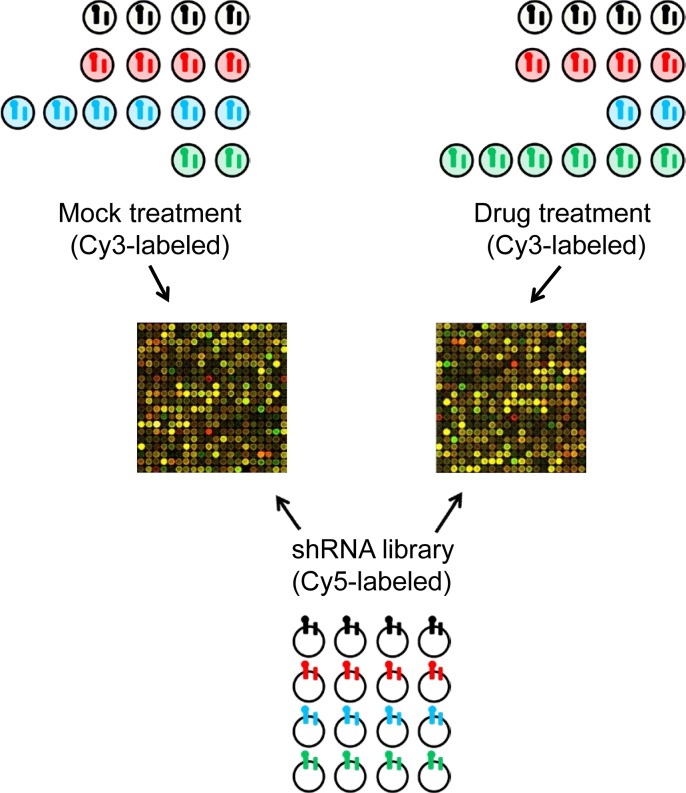
Schematic of the array protocol followed to readout the relative abundance of individual shRNAs following treatment The barcoded shRNA inserts from mock or drug treated cells are labeled with Cy3-nucleotides and used as probes in a competitive two-color hybridization with Cy5-labeled probe generated from the barcoded shRNA inserts amplified from the E. coli generated library.

To readout the relative abundance of individual shRNAs by microarray, we used vector primers to PCR amplify the shRNA and barcodes from genomic DNA prepared from human cells after selection and different treatments, and the resultant PCR product was labeled with Cy3 dye (Figure 2). We used the same primers to amplify the shRNA and barcodes from the library DNA prepared in E. coli, and used this as the common Cy5-labeled reference hybridization probe. We used competitive two-color hybridization to a custom microarray to readout the relative abundance of individual shRNAs following integration into the recipient cell line and its treatment with drug or mock treatment (Figure 2).

**Figure 3:**
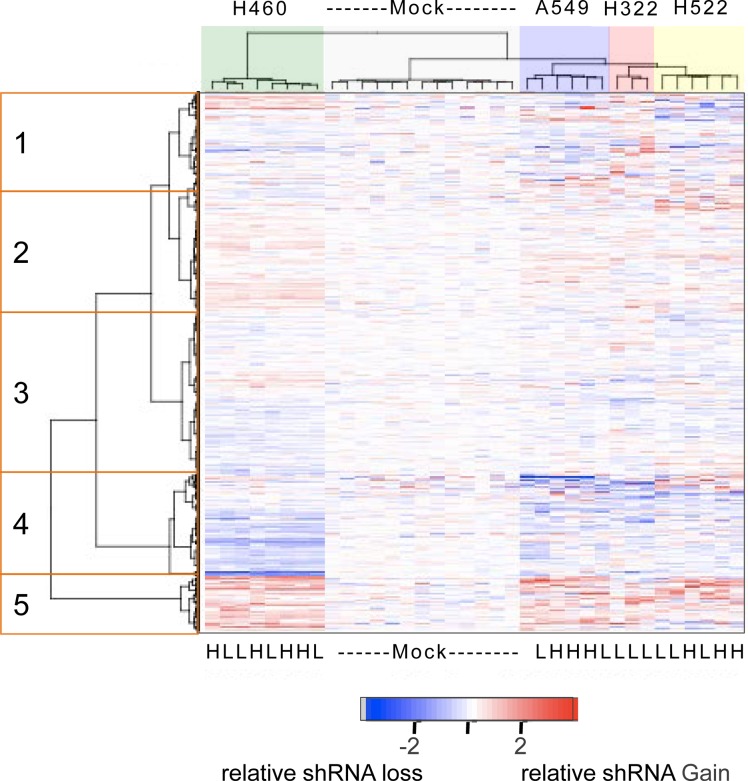
Heatmap of the relative abundance of 3,003 shRNAs in the genomic DNA isolated from mock-treated or GSK461364A-treated lung cancer cell lines Both individual shRNAs (rows) and individual cell line treatments (columns) were hierarchically clustered based on the measured abundance of shRNAs relative to the average of mock treatments. The color key indicating relative abundance is on a log2 scale. The mock treatments formed one cluster and each of the GSK461364A treatments clustered according to cell line identity but irrespective of low (IC_20_) or high (IC_80_) drug treatment (indicated by L and H at the bottom of the heatmap). The five clusters of shRNAs that are highlighted are described in the text.

Following standard microarray data processing steps including PCA analysis to exclude poor hybridizations and exclusion of hybridization probes with signals close to background levels, we obtained reliable data for 3,003 shRNAs for all hybridizations except for the three for the high-dose GSK461364A treatment of NCI-H322, which were excluded. To focus our attention strictly on the drug related effects of the individual shRNAs, we normalized the log2 fluorescent ratios to the average value of the mock treatments, so that on average the log2 ratios of mock treatments would be zero. To look at the structure of the resultant data, we performed hierarchical clustering of both the samples and the shRNAs and visualized the results with a heatmap, such that shRNAs that conferred sensitivity to GSK461364A are shown in blue, shRNAs that conferred resistance to GSK461364A are displayed in red, and shRNAs with neutral effects are shown in white (Figure 3). As expected, the individual mock treated samples appeared largely neutral, and the individual replicates for the four different cell lines all clustered by cell line identity. However, we were surprised to see that there was little if any difference between the IC_20_ or IC_80_ treatments, such that for each cell line the two treatment groups were intermingled (Figure 3). Therefore it appears that, at least for the response to GSK461364A, there is not a significant difference between high or low drug concentrations in terms of impact on the RNAi screening results.

We next turned our attention to the individual shRNAs. The visual impression (Figure 3) that a major portion of the shRNAs had an appreciable effect on response to GSK461364A was confirmed by calculating the percentage of shRNAs that were significantly gained or lost relative to mock treated cells (44%; p < 0.05). This high percentage is perhaps not too surprising given that the genes targeted by this library encode either functional cell cycle or cancer genes, or kinases [11]. The most distinct cluster of shRNAs is a set of approximately 300 shRNAs (on the bottom of the heatmap, cluster 5) that appeared to confer resistance to GSK461364A (Figure 3). This group included two shRNAs targeting *RARA*. This group behaved similarly in all four cell lines, indicating a relative uniformity of response to the individual shRNAs. The next most distinct cluster is a set of approximately 600 shRNAs that for the most part appeared to uniformly confer sensitivity to GSK461364A (cluster 4) (Figure 3). The remaining clusters included a cluster of weaker resistance shRNAs (cluster 2), weaker sensitizer shRNAs (cluster 3), and a group of shRNAs that appeared to have more variable effects on response to GSK461364A depending on the cell line (cluster 1) (Figure 3).

**Figure 4:**
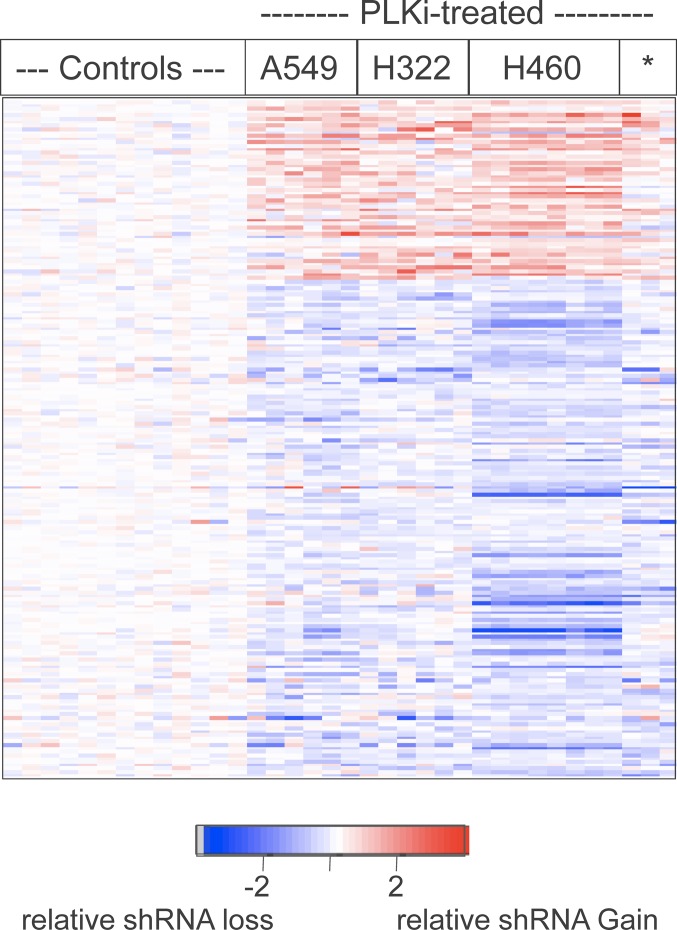
Heatmap display of the relative abundance of the 201 shRNAs chosen by SAM and independent shRNA concordance A two-class comparison (all mock treatments versus all GSK461364A treatments) by the statistical method SAM pinpointed 816 shRNAs that were significantly different. From this group of 816, 201 shRNAs showed concordance defined by targeting the same gene and showing consistently lower relative abundance or higher relative abundance. The shRNAs (rows) of the heatmap were not clustered but were ordered as a group based on relative gain or loss and within that group ordered alphabetically by gene name. Individual cell line treatments (columns) were hierarchically clustered as in Figure 4.

We used the statistical technique SAM to more rigorously determine which shRNAs were the most significant for causing resistance or sensitivity to GSK461364A. Based on the extensive similarity of shRNAs in both low and high drug concentrations, these treatments were grouped together for all cell lines into one class and compared to all the mock treatments as the other class. Using a false-discovery rate of 3.5%, 816 shRNAs were found to significantly affect the response to GSK461364A in the four lung cancer cell lines. To help rule out off target effects of individual shRNAs, we focused on the subset of the 816 significant shRNAs where there were two or more that targeted the same gene and caused the same effect (resistance or sensitivity). 201 shRNAs met this criteria, the majority of which conferred sensitivity, although not as uniformly as those conferring resistance (Figure 4).

To gain insight into the biological functions affecting by these 201 shRNAs, we examined the corresponding 97 genes using the DAVID Gene Functional Classification Tool, which utilizes multiple sources of functional annotation [13] (http://david.abcc.ncifcrf.gov). For an appropriate background set of genes for which to perform the statistical enrichment tests, we used all 693 genes that are targeted by two or more shRNAs in the library. This analysis yielded five groups of related genes that represented biological processes enriched in the 97 out of 693 genes [Table 1]. Here we briefly describe these groups and postpone addressing biological implications until the discussion section. The first group, cell surface receptor linked signal transduction, includes eight genes including *EGFR* and *IGFR1*, where the shRNAs targeting these genes enhanced the response to GSK461364A [Table 1]. The second group, comprised of 25 protein kinase genes, contained mostly sensitizer and a few resistance genes [Table 1]. The third group contains four mitotic proteins (*ANAPC5, CDC16, CDC20, CDC27*) and shRNAs targeting these mitotic genes are predicted to sensitize cancer cells to GSK461364A [Table 1]. The fourth and fifth groups are nuclear hormone receptors and protein tyrosine phosphatases [Table 1]. Of particular interest to us were the five nuclear hormone receptors involved in retinoic acid signaling: *TR2* [14], *TR4* [15], *NURR1* [16], *NOR1* [17], and the retinoic acid receptor alpha gene, *RARA*. Retinoids that activate retinoic acid receptor alpha have been shown to be safe and shown some efficacy in clinical trials with NSCLC [18]. Based on these findings and the relative safety of retinoids, we decided to further study this potential for a combination therapy with GSK461364A.

**Table 1:** Functional classification of the 97 genes associated with response to GSK461364A The DAVID Gene Functional Classification Tool was used to analyze the 97 genes targeted by the 201 shRNAs associated with response to GSK461364A. The resultant groupings and 50 genes are listed here, together with the average shRNA relative abundance (drug treatment vs. mock treatment) on a log2 scale.

Grouped Genes	Description	shRNA effect
Cell surface receptor linked signal transduction		
**CSF1R**	colony stimulating factor 1 receptor	−0.30
**DDR1**	discoidin domain receptor tyrosine kinase 1	−0.41
**EGFR**	epidermal growth factor receptor	−0.50
**GUCY2D**	guanylate cyclase 2D, membrane	−0.46
**IGF1R**	insulin-like growth factor 1 receptor	−0.28
**KIT**	proto-oncogene tyrosine-protein kinase Kit	−0.45
**TGFBR1**	transforming growth factor, beta receptor 1	−0.54
**TIE1**	tyrosine kinase with immunoglobulin-and EGF-like domains	−0.52
**Protein kinase**		
**BMX**	BMX non-receptor tyrosine kinase	−0.86
**CAMKK1**	calmodulin-dependent protein kinase kinase	−0.86
**CDC7**	cell division cycle 7 homolog (S. cerevisiae)	−0.39
**CDK7**	cyclin-dependent kinase 7	−0.34
**CSF1R**	colony stimulating factor 1 receptor	−0.29
**CSNK1G1**	casein kinase 1, gamma 1	−0.55
**CSNK2A2**	casein kinase 2, alpha prime polypeptide	−0.44
**DDR1**	discoidin domain receptor tyrosine kinase 1	−0.41
**DYRK4**	dual-specificity tyrosine-(Y)-phosphoryl. regulated kinase 4	−0.47
**EIF2AK2**	eukaryotic translation initiation factor 2-alpha kinase 2	−0.25
**ICK**	intestinal cell (MAK-like) kinase	−0.42
**LATS2**	LATS, large tumor suppressor, homolog 2 (Drosophila)	−0.54
**MAP2K6**	mitogen-activated protein kinase kinase 6	0.61
**MAP2K7**	mitogen-activated protein kinase kinase 7	−0.23
**MAP3K13**	mitogen-activated protein kinase kinase kinase 13	1.11
**MAPK10**	mitogen-activated protein kinase 10	−0.87
**MAPK14**	mitogen-activated protein kinase 14	−0.16
**MAPK15**	mitogen-activated protein kinase 15	−0.39
**MAST4**	microtubule associated serine/threonine kinase 4	−0.82
**PFKP**	phosphofructokinase, platelet	0.46
**SCYL3**	SCY1-like 3 (S. cerevisiae)	−0.86
**SGK3**	serum/glucocorticoid regulated kinase 3	0.24
**SLK**	STE20-like kinase (yeast)	−0.36
**TAOK2**	TAO kinase 2	−0.87
**TIE1**	tyrosine kinase with immunoglobulin-and EGF-like domains	−0.52
**M-phase of mitotic cell cycle**		
**ANAPC5/APC5**	anaphase promoting complex subunit 5	−0.36
**CDC16**	cell division cycle 16 homolog (S. cerevisiae)	−0.53
**CDC20**	cell division cycle 20 homolog (S. cerevisiae)	−0.56
**CDC27**	cell division cycle 27 homolog (S. cerevisiae)	−0.44
**Nuclear hormone receptor, ligand-binding**		
**NR1I3**	nuclear receptor subfamily 1, group I, member 3	−1.28
**NR2C1/TR2**	nuclear receptor subfamily 2, group C, member 1	−0.75
**NR2C2/TR4**	nuclear receptor subfamily 2, group C, member 2	−0.65
**NR2E3**	nuclear receptor subfamily 2, group E, member 3	−0.65
**NR3C2**	nuclear receptor subfamily 3, group C, member 2	−0.47
**NR4A2/NURR1**	nuclear receptor subfamily 4, group A, member 2	−1.39
**NR4A3/NOR1**	nuclear receptor subfamily 4, group A, member 3	0.71
**NR5A1**	nuclear receptor subfamily 5, group A, member 1	−0.87
**RARA**	retinoic acid receptor, alpha	0.62
**Protein-tyrosine phosphatase**		
**DCC**	deleted in colorectal carcinoma	0.79
**PTPRD**	protein tyrosine phosphatase, receptor type, D	−0.72
**PTPRF**	protein tyrosine phosphatase, receptor type, F	−0.64
**PTPRG**	protein tyrosine phosphatase, receptor type, G	−0.47

**Figure 5:**
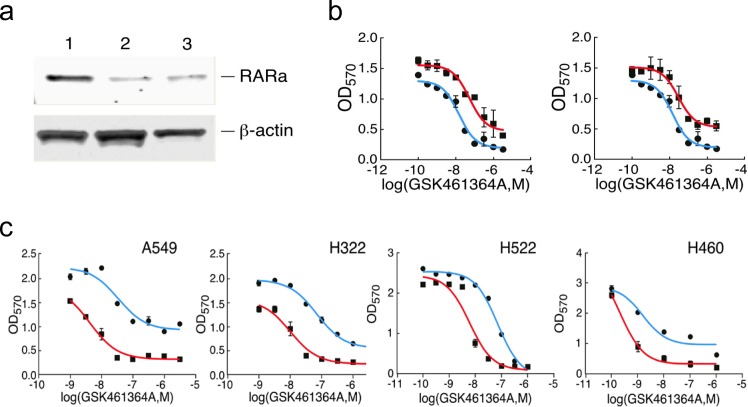
Validation of shRNAs targeting RARA and the effect of retinoids on the response of lung cancer cells to GSK461364A Panel A. Confirmation that the two shRNAs directed against *RARA* knockdown protein expression. NCI-H460 cells were stably infected with control vector (lane 1) or the two different shRNAs (lanes 2 and 3), and extracts were immunoblotted with anti-RARα antibody. Panel B. Dose-response curves showing the sensitivity of NCI-H460 cells to growth inhibition by GSK461364A that had been previously transduced with either control vector (blue line) or the two different shRNAs directed against *RARA* (red lines; each shRNA is shown in a separate graph). Panel C. Dose-response curves showing the sensitivity of four different lung cancer cell lines to varying concentrations of GSK461364A in the presence (red) or absence (blue) of 1 micromolar ATRA and 1 micromolar 9-cis-retinoic acid. 1,000-3,000 cells were plated in 96-well plates and assayed for growth inhibition as described in Methods.

We first validated that the two shRNAs pinpointed by the screen did indeed silence expression of *RARA* and confer resistance to GSK461364A when assayed individually (Figure 5). We reasoned that if silencing *RARA* could confer resistance to GSK461364A that activating its protein product with ligands could confer sensitivity. To test this idea, we examined the ability of ligands to sensitize lung cancer cells to different concentrations of PLK1 inhibitor. We found that the combination of RAR-alpha ligand (ATRA) and RXR-alpha ligand (9-cis-retinoic acid) were more potent at sensitizing cells to GSK461364A than single ligand (data not shown), in agreement with a previous report [19]. Using the combination of ATRA and 9-cis-retinoic acid, we found that the four lung cancer cell lines displayed on average a one-log increase in sensitivity to GSK461364A (Figure 5). Perhaps of greater significance in terms of therapeutic benefit is the apparent increase in number of cells killed at higher doses of GSK461364A for three of the four lung cancer cell lines (Figure 5). Encouraged by these results, we next examined whether ATRA and 9-cis-retinoic acid potentiated cancer cell inhibition by GSK461364A by enhancing its previously demonstrated ability to cause mitotic arrest and apoptosis. After three days of treatment, we examined A549 lung cancer cells by flow cytometry to determine their position in the cell cycle and by immunofluorescence using antibodies to the mitotic marker histone H3 and apoptotic marker active caspase-3 to measure mitosis-associated apoptosis. This analysis showed that ATRA and 9-cis-retinoic acid enhanced the ability of GSK461364A to induce cell cycle arrest in G2/M and also significantly increased the percentage of cells undergoing mitotic-associated apoptosis, from less than 5% for GSK461364A alone to 32% of the cells (Figure 5). Thus ATRA and 9-cis-retinoic acid does not appear to change the cellular mechanism of action of GSK461364A but instead act as sensitizers. As discussed below, these results establish retinoids are interesting candidates for combination therapy strategies with GSK461364A.

## DISCUSSION

Our results indicate that shRNA screening is an effective method for identifying the biological processes that are potentially important in anti-cancer drug action as well as for identifying combination cancer drug strategies. One of the most critical steps in our analysis of the array-based screening results for the four different lung cancer cell lines was to normalize the individual shRNA readouts for the drug treatments to the average value of the mock treatments. This step eliminated much of the cell line variability that existed for individual shRNAs and enabled us to determine that a substantial portion of shRNAs in the library did affect response to the PLK1 inhibitor GSK461364A. As our library was focused on kinases and cell cycle genes, whether such a high percentage of biologically active shRNAs would be observed in a true genome-wide shRNA screen is far from certain. Nevertheless, by focusing on the statistically significant shRNA changes and by filtering against off-target effects by requiring two or more significant shRNAs targeting the same gene to have the same effect, we were able to identify a set of 97 genes that were enriched for important biological functions that are likely important mediators of the ability of GSK461364A to inhibit cancer cell proliferation. Amongst these functions was cell-surface tyrosine kinase receptor signaling, including the *EGFR* and *IGFR1* genes. It was somewhat surprising that our results predicted that silencing *EGFR* or *IGFR1* would have an effect in our cell line panel since two cell lines harbor mutant *KRAS* and the current paradigm is that mutant *KRAS* obviates any dependency upon upstream receptor tyrosine kinase signaling. However, phenotypic effects of inhibiting these two genes in mutant-*KRAS* NSCLC cell line have been observed, suggesting that this paradigm may not always accurately reflect cancer biology [20]. Another key functional group included the anaphase complex proteins CDC20, CDC16, CDC27, and APC5. That loss-of-function of these proteins would enhance the effects of inhibiting polo-kinase 1 is in line with the notion that one PLK1 primary function is to promote the activation of the anaphase complex during mitosis [6].

Another key group were nuclear hormone receptors that affect retinoic acid signaling, including retinoic acid receptor alpha (*RARA*). We picked this group for follow-up studies based on the possibility of combining GSK461364A with relatively non-toxic retinoids, which had previously been established as being tolerated and potentially effective in NSCLC [18]. Two shRNAs designed against *RARA* that were pinpointed by the array study were validated for their ability to silence *RARA* expression and confer resistance to GSK461364A. Conversely, we showed that activating *RARA* with retinoids induced increased sensitivity by an order of magnitude to GSK461364A, an effect seen in all four lung cancer cell lines. These results provide a strong rationale for including a retinoid arm in future clinical trials of PLK1 inhibitors.

In conclusion, our results provide further evidence for the utility of shRNA pooled screening in designing combination therapy strategies for cancer drugs. Several aspects of pooled shRNA screening are being further optimized and should lead to even more effective ability to uncover combination strategies. These optimizations include the use of larger libraries that encompass more of the whole genome [21] and next generation sequencing instead of microarrays for a more sensitive and robust readout of relative shRNA abundance [22].

## METHODS

### RNAi screen and computational analysis

The RNAi screen was performed according to the protocol published by Silva et al. using multiplex Agilent microarrays [11]. Probe intensity files for the microarrays were generated from Agilent’s Feature Extraction software, and we used the column “gMeanSignal” as test channel signal, and column “rMeanSignal” as reference channel signal. Probes from shRNAs not used in our library were used as negative probes for background estimation. The array contained two types of probes: barcode probes (60 mers) and half hairpin probes (21 mers). As these probes behaved differently, the barcode probe set and half hairpin probe set; they were processed separately during the background filter and normalization steps. We performed spatial correction to remove spatial effects resulting from uneven washing, evaporation edge effect and so on. The spatial correction used a window of 300 probes. Within each window, the intensity of the inside probes was scaled to make sure the median probe intensity in the window was equal to the median on the whole array. We then applied a background filter: for each probe set, we calculated the median intensity of background probes in each channel for each sample in the mock treatment group. These medians are taken to be estimations of the background. Next, we removed probes from the dataset whose intensity was less than 1.5 times of background in red or green channel in more than half of the samples in the mock treatment group. We then applied global loess normalization to remove the imbalance between the red and green channels, using the R package limma (version 2.8.1) and the function “normalizeWithinArrays”. The parameter “method” in that function is chosen as “loess”. After normalization, we obtain a log (base 2) ratio between green channel and red channel for each probe.

Following normalization, we processed the shRNAs that have both barcode probe and half hairpin probe printed on the array. To collapse the data from the probe level into the shRNA level, one choice would be to take the mean of the probe ratios. But as the two types of probes are different in probe length and nucleotide sequence, they might have different measurement errors and qualities. If we take the average, the good probe might be compromised by the relatively bad probe. So we sought to use the probe with higher signal to noise to represent the shRNA. To determine which probe has higher quality, we define an index SNR (signal to noise ratio) as:

$$\text{SNR}=\frac{\text{between group variability}}{\text{within-group variability}}=\frac{\frac{\sum_{i=1}^{K}n_{i}{\left(\bar{X_{i.}}-\bar{X}\right)}^{2}}{K-1}}{\frac{\sum_{i=1}^{K}\sum_{j=1}^{n_{i}}{\left(\bar{X_{ij}}-\bar{X_{i.}}\right)}^{2}}{N-K}}$$

Where ${\bar{X}}_{i}$ denotes the sample mean in the *i^th^* group indicator, *n_i_* is the number of observations in the *i^th^* group, $\bar{X}$ denotes the overall mean of the data, *x_ij_* is the *j^th^* observation in the *i^th^* group out of *K* groups and *N* is the overall sample size.

Following normalization of the log2 ratios for the mock, IC_20_ and IC_80_ treatments to the average of the mock treatments, the distributions of the ratios for the drug treatments for the different cell lines were adjusted using normalize.quantile.robust in the R library “preprocessCore.” Clustering and heatmaps were performed using heatmap.2 in R and SAM was performed using the “siggenes” R package.

### Cell biology assays

GSK461364A treatment was for three days followed by a washout of the compound and recovery in growth medium for three to seven days. GSK461364A was removed from plates by three washes with 20% FBS-supplemented complete medium. To test for combination treatment of retinoids with GSK461364A, 1,000-3,000 cells were plated in clear-bottom 96-well plates (Corning). Following retinoid (1 micromolar All-trans-retinoic acid, ATRA; 1 micromolar 9-cis-retinoic acid, 9cRA, Sigma) pre-treatment for 96 hours, the cells were cultured in the presence of retinoids and GSK461364A or DMSO vehicle for 72 hours. The drugs were then removed by washing 3X with cell culture medium supplemented with 20% FBS, followed by post-treatment with retinoids or DMSO for an additional 72 hours. The cells were stained with MTT [3-(4,5-dimethylthiazol-2-yl)-2,5-diphenyltetrazolium bromide] colorimetric reagent and solubilized following the manufacturer’s instructions (Roche) and subjected to automated plate reading (Victor3, PerkinElmer) at OD 595nm following the manufacturer’s instructions. To assay for mitotic arrest and apoptosis, A549 cells were plated on cover slips followed by treatment of 1 micromolar retinoids plus GSK461364A (IC_20_), retinoids alone, or GSK461364A alone, or DMSO. 72 hours post drug treatment, the cells were ethanol-fixed and propidium iodide stained, followed by flow cytometry analysis for DNA content distribution. In parallel, 72 hours post drug treatment, the cells were fixed in 3.7% paraformaldehyde, permeabilized in 0.1% Triton X-100/PBS, blocked in 1% BSA in PBS, then sequentially stained with anti-histone H3 (Ser10, Abcam), 16 hours at 4^o^ C, and anti-active caspase 3 (Cell Signaling), 16 hours at 4^o^ C. Following incubation with fluorescently labeled secondary antibodies (Alexa488 for H3 and Alexa 568 for Casp3, Molecular Probes), the cells were counter-stained with DAPI, mounted, sealed, and photographed using AxioVision (Zeiss, 20x).

**Figure 6:**
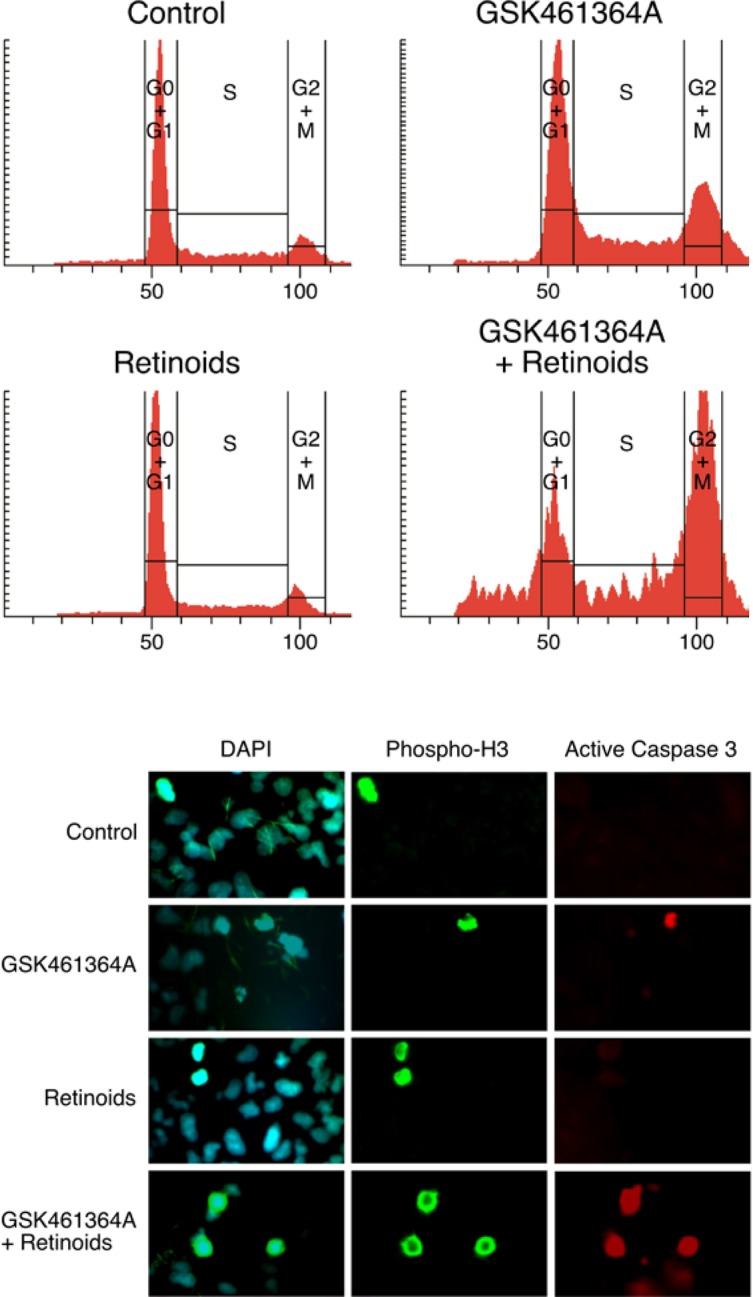
Characterization of the cell cycle arrest and mitosis-associated apoptosis induced by GSK461364A and retinoids Panel A. Flow cytometric analysis of DNA content after three-day treatment of A549 cells with either mock treatment, retinoids alone (ATRA and 9-cis-retinoic acid both at 1 micromolar), GSK461364A alone [10 nM], or GSK461364A [10nM] plus retinoids (ATRA and 9-cis-retinoic acid both at 1 micromolar). A549 cells were ethanol-fixed and stained with propidium iodide. Panel B. The effects of ATRA on the ability of GSK461364A to induce mitosis-associated apoptosis three days post-treatment. The concentration of retinoids used was ATRA and 9-cis-retinoic acid both at 1 micromolar and for GSK461364A was 10 nM. A549 cells were fixed and reacted sequentially with anti-phosphohistone H3 (phospho-Ser10; Abcam), anti-active Caspase 3 (Cell Signaling), and secondary antibodies with fluorescent labels Alexa 488 (for anti-phosphohistone H3) and Alexa 568 (anti-active Caspase3) (Molecular Probes). The cells then were counter-stained with DAPI, mounted, sealed, and photographed using a Zeiss AxioVision (20x). 200 cells were scored for each condition, and the background percentage of mitotic-associated apoptotic cells for mock treatment was 1%, 4% for treatment with 10 nM GSK461364A, 2% for treatment with retinoids alone, and 33% for treatment with both GSK461364A and retinoids.
